# Supplementary material for: 99mTc-Labeled FAPI SPECT Imaging in Idiopathic Pulmonary Fibrosis: Preliminary Results
Source: Pharmaceuticals (Basel). 2023 Oct 9;16(10):1434. doi: 10.3390/ph16101434 (PMC10610005; doi:10.3390/ph16101434)
Supplement: Supplementary file 1 [file pharmaceuticals-16-01434-s001.zip › pharmaceuticals-2535732-supplementary.pdf]

pharmaceuticals

# Supplementary Materials: $^{99m}$Tc-Labeled FAPI SPECT Imaging in Idiopathic Pulmonary Fibrosis: Preliminary Results

Yu Liu[1], Qian Zhang[2], Yuwei Zhang[1], Jingnan Wang[1], Yitian Wu[1,4], Guangjie Yang[1], Jiyun Shi[3], Fan Wang[3], Zuojun Xu[2*] and Hongli Jing[1*]

**Supplementary information:**

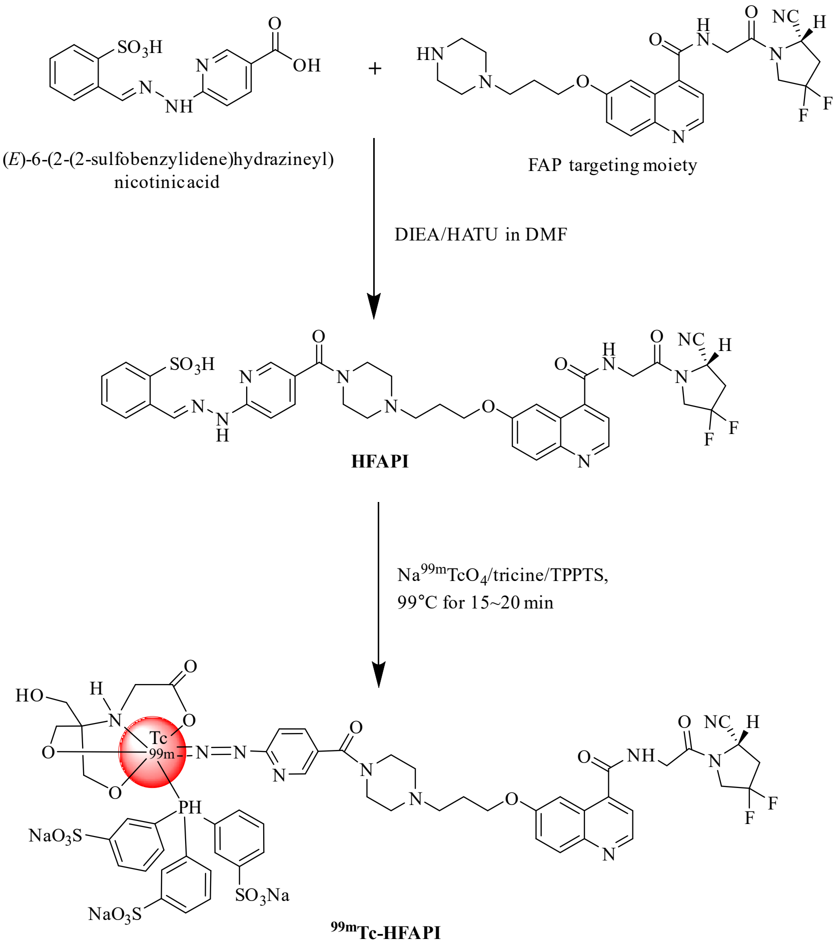


**Figure S1.** Chemical synthesis and $^{99m}$Tc radiolabeling of HFAPI.

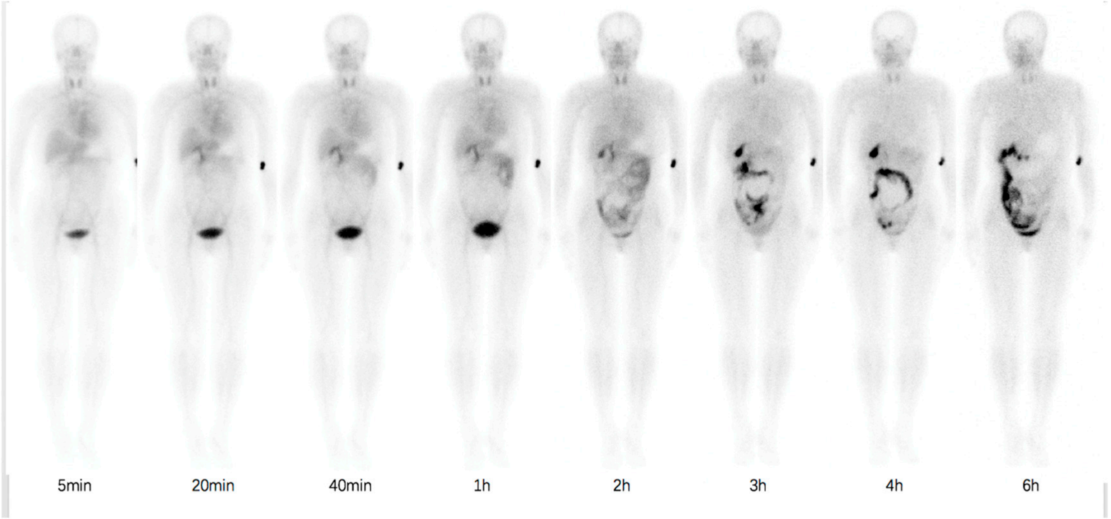


**Figure S2.** A representative whole-body anterior projection images (A) at different time points (5 min, 20 min, 40 min, 1 h, 2 h, 3 h, 4 h, 6 h) after intravenous injection of $^{99m}$Tc-HFAPI and SPECT/CT MIP (B) of a 56-year-old female ovarian cancer patient without lung affected. Note there is no evident uptake in the healthy lung region.
